# Is Dementia More Fatal Than Previously Estimated? A Population-based Prospective Cohort Study

**DOI:** 10.14336/AD.2018.0123

**Published:** 2019-02-01

**Authors:** Jong Bin Bae, Ji Won Han, Kyung Phil Kwak, Bong Jo Kim, Shin Gyeom Kim, Jeong Lan Kim, Tae Hui Kim, Seung-Ho Ryu, Seok Woo Moon, Joon Hyuk Park, Jong Chul Youn, Dong Young Lee, Dong Woo Lee, Seok Bum Lee, Jung Jae Lee, Jin Hyeong Jhoo, Ki Woong Kim

**Affiliations:** ^1^Department of Psychiatry, Seoul National University College of Medicine, Seoul, Korea.; ^2^Department of Neuropsychiatry, Seoul National University Bundang Hospital, Gyeonggido, Korea.; ^3^Department of Psychiatry, Dongguk University Gyeongju Hospital, Gyeongju, Korea.; ^4^Department of Psychiatry, Gyeongsang National University, School of Medicine, Jinju, Korea.; ^5^Department of Neuropsychiatry, Soonchunhyang University Bucheon Hospital, Bucheon, Korea.; ^6^Department of Psychiatry, School of Medicine, Chungnam National University, Daejeon, Korea.; ^7^Department of Psychiatry, Yonsei University Wonju Severance Christian Hospital, Wonju, Korea.; ^8^Department of Psychiatry, School of Medicine, Konkuk University, Konkuk University Medical Center, Seoul, Korea.; ^9^Department of Psychiatry, School of Medicine, Konkuk University, Konkuk University Chungju Hospital, Chungju, Korea.; ^10^Department of Neuropsychiatry, Jeju National University Hospital, Jeju, Korea.; ^11^Department of Neuropsychiatry, Kyunggi Provincial Hospital for the Elderly, Korea.; ^12^Department of Neuropsychiatry, Seoul National University Hospital, Seoul, Korea.; ^13^Department of Neuropsychiatry, Inje University Sanggye Paik Hospital, Seoul, Korea.; ^14^Department of Psychiatry, Dankook University Hospital, Cheonan, Korea.; ^15^Department of Neuropsychiatry, Kangwon National University Hospital, Korea.; ^16^Department of Brain and Cognitive Science, Seoul National University College of Natural Sciences, Seoul, Korea.

**Keywords:** dementia, Alzheimer’s disease, mortality, death, survival

## Abstract

Dementia increases the risk of mortality (ROM) in the elderly and estimates of hazard ratio (HR) of dementia for mortality have ranged from 1.7 to 6.3. However, previous studies may have underestimated ROM of dementia due to length bias, which occurs when failing to include the persons with rapidly progressive diseases, who died before they could be included in the study. This population-based prospective cohort study conducted on 6,752 randomly sampled Koreans, aged 60 years or older (the Korean Longitudinal Study on Cognitive Aging and Dementia). Cognitive disorders were evaluated at baseline and 2-year follow-up using the Korean version of the Consortium to Establish a Registry for Alzheimer’s Disease Assessment Packet (CERAD-K), and prevalent and incident cases of dementia were identified. The participants’ deaths were confirmed through the National Mortality Database of Statistics Korea. We compared the ROM between prevalent and incident dementia, and estimated HR of dementia for mortality using Cox proportional hazards model. Of the 5,097 responders to the 2-year follow-up assessment, 150 participants had dementia from the baseline (prevalent dementia), and 95 participants developed dementia during the 2-year follow-up period (incident dementia). The ROM of participants with incident dementia was about 3 times higher than the ROM of those with prevalent dementia (HR = 3.04, 95% confidence interval [CI] = 1.34-6.91). Compared to cognitively normal participants at both the baseline and 2-year follow-up assessments, the ROM of those with incident dementia approximately 8 times higher (HR = 8.37, 95 % CI = 4.23-16.54). In conclusion, the ROM of dementia using prevalent cases was underestimated due to length bias, and dementia may be much more fatal than previously estimated. In clinical settings, the ROM of dementia warrants the attention of physicians, particularly in recently incident dementia cases.

The number of deaths due to dementia more than doubled, in the period from 2000 to 2015, making it the seventh leading cause of death, globally, and the third leading cause of death, in high-income countries [1]. Dementia killed about 1.6 million people in 2015; this accounts for about 3% of the 56.4 million deaths, worldwide, in that year [1].

**Figure 1.**
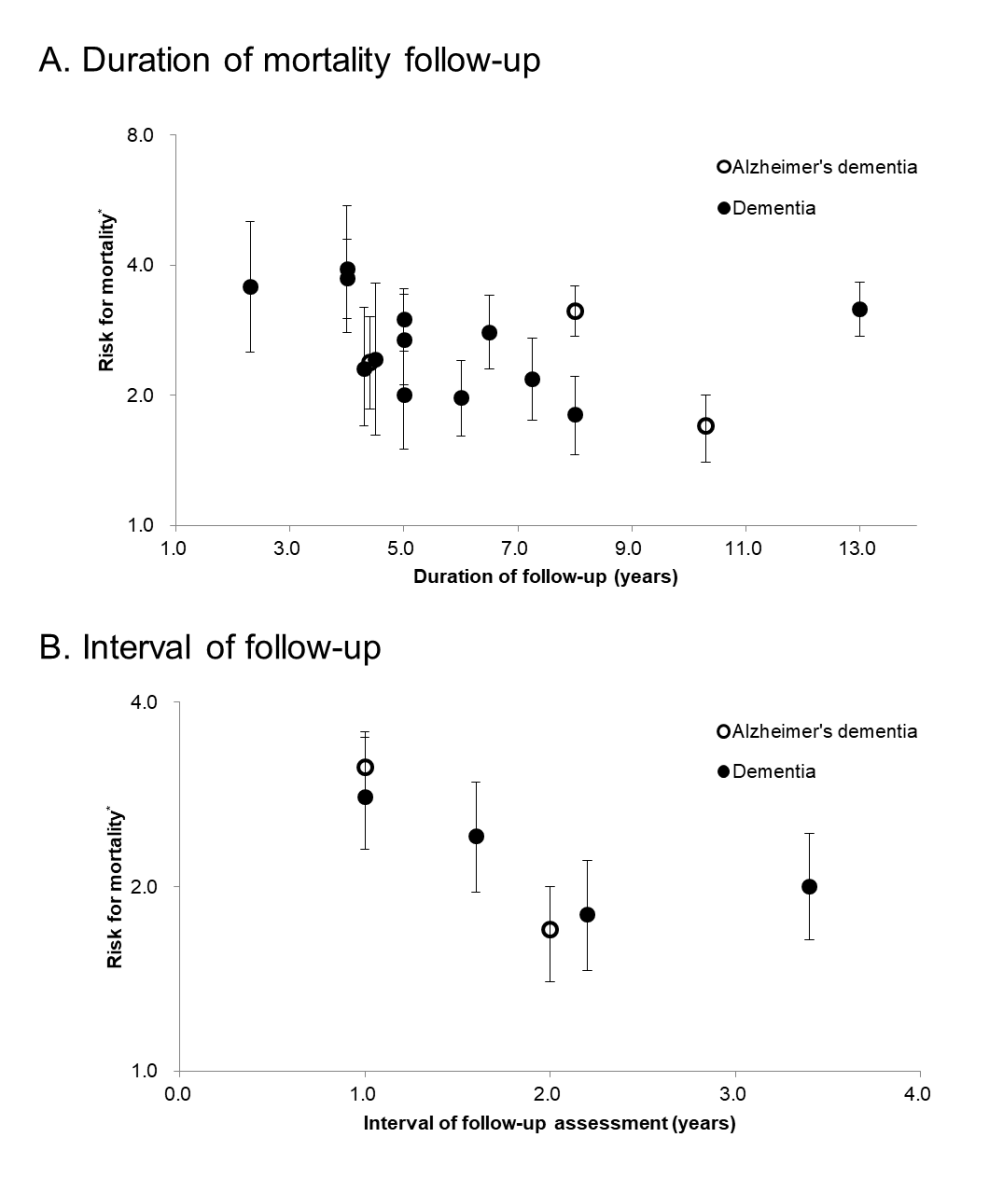
**The estimated risks of mortality of dementia according to the interval and duration of follow-up**. ^*^Estimated using Cox proportional hazard models and compared to non-demented participants; the error bars indicate 95% confidence. The references are as follows: [2-5, 7-9, 12-20].

Previously conducted population-based cohort studies consistently reported that dementia [2-20] and mild cognitive impairment (MCI) [6, 10, 11, 16, 21-23] increased the risk of mortality (ROM) in elderly people. However, the ROM of dementia and MCI might have been underestimated in those studies, due to a number of reasons. First, most of the studies estimated the ROM of dementia and MCI in prevalent cases. Of the 19 studies on the ROM of dementia published from 1998 to 2017 [2-20], only 6 were conducted on incident cases [13-18]. Of the 7 studies on the ROM of MCI, published from 2003 to 2017 [6, 10, 11, 16, 21-23], only 1 was conducted on incident cases [22]. In the studies that employed prevalent cases only, the ROM could have been underestimated due to length bias, because prevalent cases might not include the persons with rapidly progressive diseases, who died before they could be included in the study [24-27]. Second, many of those studies estimated the ROM of dementia by employing non-demented elderly people, instead of cognitively normal elderly people as the reference group [2-4, 15, 16, 18-20, 24, 28]. Since MCI also increased the ROM [22, 29, 30], the ROM of dementia could have been underestimated if the non-demented reference group included cases of MCI. However, only 3 studies, till date, have estimated the ROM of dementia, relative to a reference group comprising those without cognitive impairment [6, 10, 11]. Six studies that investigated the ROM of dementia, using incident cases, included MCI in their reference groups. Third, the ROM might have been underestimated in some studies due to their designs. The ROM of dementia tended to decrease as the follow-up interval increased (Fig. 1); the hazard ratio (HR) for mortality due to dementia was over 2, when the interval of the follow-up assessment was under 2 years [14, 16, 18], and below 2, when the interval of the follow-up assessment was 2 years or more [13, 15, 17].

In the present 4-year nationwide population-based prospective cohort study, we hypothesized that if length bias affects the estimates of ROM of dementia, the ROM of incident dementia would be higher than that of prevalent dementia. Furthermore, in order to establish the unbiased ROM of dementia, we estimated it in incident cases with dementia, and employed cognitively normal individuals as the reference group.

**Figure 2.**
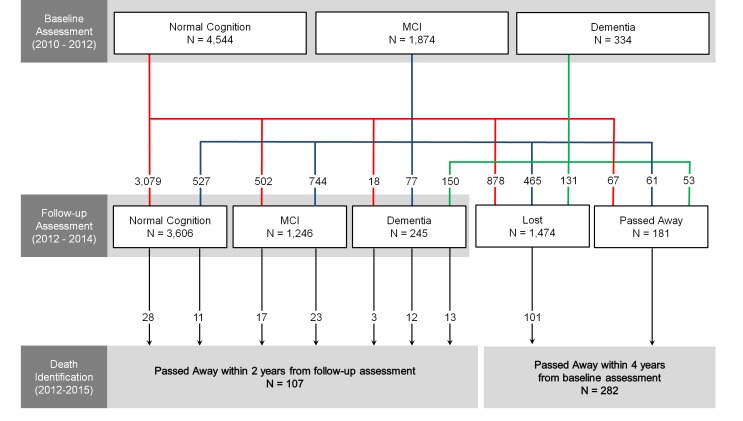
**The flowchart of the mortality analysis in the Korean Longitudinal Study on Cognitive Aging and Dementia**. MCI = mild cognitive impairment.

## MATERIALS AND METHODS

### Participants

This study was a part of the Korean Longitudinal Study on Cognitive Aging and Dementia (KLOSCAD) [32]. In the KLOSCAD, we randomly sampled 30 villages and towns from 13 specific districts across South Korea (hereafter, Korea). Using residential rosters and data on residents aged 60 years and above, we randomly selected 10% of the elderly adults from urban areas and 20% from rural areas. In the baseline assessment, which was conducted from November 2010 through October 2012, 6,818 (53.7%) individuals participated. 6,752 were included in the current analysis, after 3 individuals who had mental retardation, 2 in whom the diagnosis for cognitive disorders due to a comorbid major depressive disorder was uncertain, and 61 who did not provide their personal identification numbers, that are necessary for the identification of death at the National Mortality Database of Statistics Korea, were excluded. Follow-up assessments have been conducted every two years, with the first follow-up assessment conducted between November 2012 and October 2014. 5,097 participants completed the 2-year follow-up assessment, 181 passed away during the follow-up period, and 1,474 refused to undergo the follow-up assessment (Fig. 2). This study’s protocol was approved by the Institutional Review Board of Seoul National University Bundang Hospital. All subjects were fully informed of the study protocol. All subjects provided written informed consent, which was signed by either the subjects or their legal guardians.

### Assessments and Diagnosis

In the KLOSCAD, geriatric neuropsychiatrists with expertise in dementia research conducted a face-to-face standardized diagnostic interview, as well as physical and neurological examinations, using the Korean version of the Consortium to Establish a Registry for Alzheimer’s Disease Assessment Packet (CERAD-K) Clinical Assessment Battery and the Korean version of the Mini International Neuropsychiatric Inventory [33]. Comorbid medical illnesses were evaluated using the Cumulative Illness Rating Scale (CIRS)[34] and the vascular components of cognitive impairment using the Modified Hachinski Ischemic Score (MHIS) [35]. Trained research neuropsychologists administered the CERAD-K Neuropsychological Assessment Battery (CERAD-K-N), Digit Span Test, and Frontal Assessment Battery. The CERAD-K-N consists of 9 neuropsychological tests: The Verbal Fluency Test, 15-item Boston Naming Test, Mini Mental Status Examination, Word List Memory Test, Constructional Praxis Test, Word List Recall Test, Word List Recognition Test, Constructional Recall Test, and Trail Making Test. A panel of research neuropsychiatrists determined the final diagnosis of each participant. Dementia and depressive disorders were diagnosed according to the criteria of the Diagnostic and Statistical Manual of Mental Disorders, Fourth Edition [36], and, further, Alzheimer’s disease (AD) was diagnosed according to the criteria of the National Institute of Neurological and Communicative Disorders and Stroke and the Alzheimer’s Disease and Related Disorders Association [37]. We diagnosed MCI according to the consensus criteria from the International Working Group on MCI [38]. We ascertained the presence of objective cognitive impairment if a participant performed -1.5 standard deviation or below of the age-, gender-, and education-adjusted norms in any of the 11 neuropsychological tests [39]. We defined normal cognition (NC) as the state of having no cognitive disorders (MCI or dementia) and other psychiatric or neurologic disorders.

### Identification of Death

The deaths of participants were identified from the National Mortality Database of Statistics Korea using personal identification numbers. In Korea, deaths are reported to the corresponding local governments, and included in a national database of deaths in the country, which is managed by Statistics Korea. This database provides the date, place, and cause of death, as confirmed by a physician [40]. In the case of those who responded to the 2-year follow-up assessment, we identified their death within 2 years from their 2-year follow-up assessment. In the non-responders to the 2-year follow-up assessment, we identified their death within 4 years from their baseline assessment.

### Statistical Analysis

We compared the continuous variables, between the groups, using an independent sample t-test and one-way analysis of variance, and the categorical variables using the χ^2^ test. We investigated the association of cognitive disorders with the ROM using Cox proportional hazard models. We adjusted for potential confounding factors such as age, sex, years of education, smoking status, alcohol consumption status, MHIS, CIRS score and presence of depressive disorders, because these factors were associated with the ROM in the elderly. The appropriateness of the models was checked graphically by plotting the log[-log(survival)] curves versus log(time). First, we estimated the 4-year ROM of the prevalent cases, at the baseline assessment (NC, MCI, and dementia), using multivariate Cox proportional hazard models that adjusted potential confounding factors. This analysis included 6,752 individuals who participated in the baseline assessment, regardless of their participation in the follow-up assessment. Second, we compared the ROM between the prevalent and incident cases of dementia, AD and MCI, using multivariate Cox proportional hazard models that adjusted potential confounding factors. This analysis included 5,097 individuals who participated in both the baseline and 2-year follow-up assessments. Third, individuals with NC at both the assessments were assigned to the reference group, and we estimated the ROM in prevalent and incident cases of dementia and MCI, using multivariate Cox proportional hazard models, which adjusted potential confounding factors. Fourth, we constructed Kaplan-Meier survival plots and performed log-rank tests to estimate the cumulative survivals of the cases of prevalent and incident dementia and MCI. All the statistical analyses were performed using SPSS Statistics 20, Release Version 20.0.0 (SPSS, Inc., 2011, Chicago, IL).

**Table 1:** Baseline characteristics of the responders and non-responders, at the 2-year follow-up assessment.

Baseline characteristics	Responders	Non-responders	Statistics*	
			T or χ^2^	p
Normal cognition				
Number	3599	878		
Age (years ± SD)	68.9 ± 6.1	69.2 ± 6.4	-1.108	0.268
Sex (men, %)	44.8	45.7	0.197	0.657
Education (years ± SD)	8.9 ± 5.3	8.1 ± 5.3	4.312	< 0.001
Smoking status (yes, %)†	31.0	30.8	0.004	0.950
Alcohol consumption (yes, %)†	34.2	33.8	0.049	0.824
MHIS (score ± SD)	0.7 ± 0.9	0.6 ± 0.7	1.511	0.131
CIRS score (score ± SD)	4.3 ± 2.8	3.9 ± 2.5	4.176	< 0.001
Depression (yes, %)‡	2.0	3.1	3.059	0.080
Mild cognitive impairment				
Number	1348	465		
Age (years ± SD)	71.8 ± 6.7	72.4 ± 8.0	-1.352	0.177
Sex (men, %)	38.5	35.5	1.300	0.254
Education (years ± SD)	6.9 ± 5.0	5.7 ± 4.8	4.590	< 0.001
Smoking status (yes, %)†	26.9	24.6	0.872	0.350
Alcohol consumption (yes, %)†	28.3	24.5	2.354	0.125
MHIS (score ± SD)	0.9 ± 1.1	0.9 ± 1.2	-0.919	0.358
CIRS score (score ± SD)	4.9 ± 2.9	4.6 ± 3.0	1.474	0.141
Depression (yes, %)‡	5.8	5.0	0.392	0.531
Dementia				
Number	150	131		
Age (years ± SD)	77.9 ± 7.9	79.7 ± 7.7	-1.957	0.051
Sex (men, %)	32.0	24.4	1.969	0.161
Education (years ± SD)	3.8 ± 4.5	3.3 ± 4.4	0.800	0.424
Smoking status (yes, %)†	20.4	22.2	0.123	0.726
Alcohol consumption (yes, %)†	13.5	5.5	4.522	0.033
MHIS (score ± SD)	1.5 ± 1.9	1.7 ± 2.6	-1.017	0.310
CIRS score (score ± SD)	4.9 ± 2.8	5.7 ± 3.7	-1.972	0.050
Depression (yes, %)‡	12.2	11.0	0.087	0.776
All				
Number	5097	1474		
Age (years ± SD)	70.0 ± 6.6	71.1 ± 7.7	-5.322	< 0.001
Sex (men, %)	42.8	40.6	2.286	0.131
Education (years ± SD)	8.2 ± 5.4	6.9 ± 5.3	8.615	< 0.001
Smoking status (yes, %)†	29.6	28.2	0.986	0.321
Alcohol consumption (yes, %)†	32.0	28.6	5.881	0.015
MHIS (score ± SD)	0.7 ± 1.0	0.8 ± 1.2	-2.191	0.029
CIRS score (score ± SD)	4.5 ± 2.8	4.3 ± 2.9	2.323	0.020
Depression (yes, %)‡	3.3	4.3	2.971	0.085

SD = standard deviation, MHIS = Modified Hachinski Ischemic Score, CIRS = Cumulative Illness Rating Scale

* Continuous variables were compared using a t-test and categorical variables were compared using χ^2^ tests

† Smoked or drank alcohol within the past one year

‡ Diagnosed as having major or minor depressive disorders

## RESULTS

Of the 6,752 individuals who participated in the baseline assessment, 181 passed away within 2 years. In total, 5,097 participants responded to the 2-year follow-up assessment and 1,474 refused to participate in the follow-up assessment. At the baseline assessment, MCI and dementia were less prevalent in those who participated in the 2-year follow-up assessment compared to those who did not (26.4% versus 31.6% for MCI, 2.9% versus 8.9% for dementia, p < 0.001, Chi square test). The responders were younger, more educated, and likelier to be alcohol drinkers, and had higher CIRS scores; however, their MHIS was lower than that of the non-responders (Table 1).

Of those who responded to the 2-year follow-up assessment, 107 passed away within 2 years from the follow-up assessment. Among the non-responders, 181 and 99 passed away before and after the 2-year follow-up assessment, respectively. Compared to the participants with NC, at the baseline assessment, those with MCI showed approximately a 1.5-fold higher risk of 4-year mortality (HR = 1.49, 95% confidence intervals [CI] = 1.16-1.92) and those with dementia showed about a 2.7-fold higher risk of 4-year mortality (HR = 2.67 95% CI = 1.90-3.74). The HR for 4-year mortality of dementia was reduced to about 2.14 (95% CI 1.57-2.90) when we employed the non-demented people, i.e., people with NC or MCI, as the reference group.

**Table 2:** Comparison of the mortality risks between prevalent and incident cases.

Diagnosis	Type	Number of participants	Person-years	Number of deaths	HR (95% CI)*
Dementia	Prevalent cases	150	251.8	13	1.00
	Incident cases	95	158.5	15	3.04 (1.34 - 6.91)
Alzheimer’s disease	Prevalent cases	117	201.5	9	1.00
	Incident cases	67	118.0	9	2.90 (1.06 - 7.97)
Mild cognitive impairment	Prevalent cases	744	1285.0	23	1.00
	Incident cases	502	848.5	17	1.24 (0.65 - 2.34)

HR = hazard ratio; CI = confidence intervals

* Adjusted by age, educational level, sex, alcohol consumption, smoking status, Cumulative Illness Rating Scale score, Modified Hachinski Ischemic Score and the presence of a depressive disorder

Of 5,097 participants who responded to the 2-year follow-up assessment, 150 participants had dementia in both the baseline and follow-up assessments (hereafter, prevalent dementia), and 95 participants who were not demented in the baseline assessment converted to dementia in the follow-up assessment (hereafter, incident dementia). The participants with incident dementia showed about a 3-fold higher risk of 2-year mortality than those with prevalent dementia (Table 2). Compared to the participants with prevalent dementia, those with incident dementia were more educated (3.79 ± 4.49 vs 5.58 ± 5.20, p = 0.005), and had fewer smoker (33.0% vs 20.4%, p = 0.030) and higher CIRS scores (4.94 ± 2.82 vs 6.19 ± 3.42, p = 0.003) at the baseline assessment. At the 2-year follow-up assessment, however, the participants with incident dementia had more very mild (clinical dementia rating [CDR] = 0.5) or mild (CDR = 1) cases, relative to those with prevalent dementia (90.0% vs 71.0%, p = 0.001). There were no significant differences in terms of the other demographic and clinical characteristics, including age, sex, MIHS, alcohol consumption status, diagnosis of depression, and the distribution of the dementia subtypes (p > 0.1), between the groups. The distributions of the dementia subtypes were also comparable between the prevalent and incident cases; 74% of the prevalent dementia and 69% of the incident dementia cases had AD (χ^2^= 1.650, p = 0.199). The participants with incident AD also showed about a 3-fold higher risk of 2-year mortality than those with prevalent AD. The participants with incident dementia, who passed away within 2 years from the diagnosis, were more likely to be male (p = 0.043) and alcohol drinkers (p = 0.002) than those who survived for 2 years after the diagnosis. However, the other demographic and clinical characteristics, including age, years of education, smoking status, CIRS score, MIHS, and diagnosis of depression were comparable between the 2 groups. Although the 2-year ROM of incident MCI was also higher than that of prevalent MCI, the difference was statistically insignificant.

**Figure 3.**
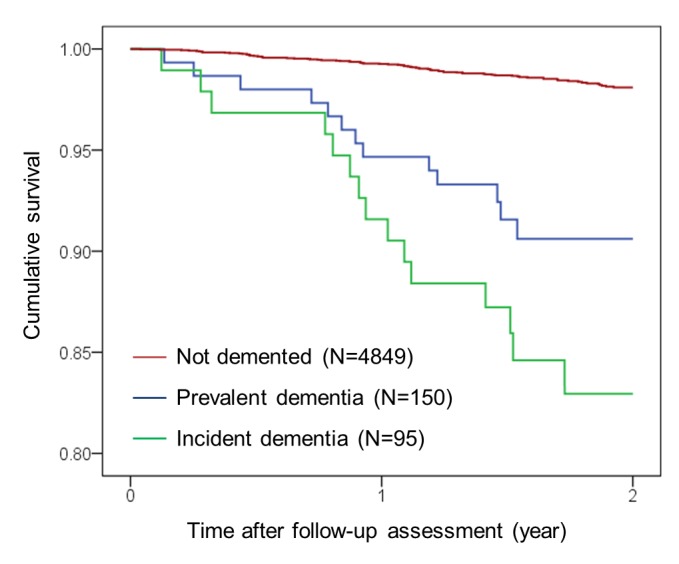
Kaplan-Meier survival curves of the prevalent and incident cases with dementia.

At the 2-year follow-up assessment, 18 and 77 individuals, who had NC and MCI, respectively, at the baseline assessment, were converted to dementia, and 502 participants who had NC, at the baseline assessment, were converted to MCI. Of the participants with MCI, at the baseline, 527 cases reverted to NC (hereafter, reverted MCI) and in 722, the status did not change (hereafter, stable MCI). Compared with the participants who had NC at both the baseline and 2-year follow-up assessments (hereafter, stable NC), those with incident dementia showed about an 8-fold increased risk of 2-year mortality (HR = 8.37 95 % CI = 4.23-16.54). The mortality HR of incident dementia was 8.20 (95% CI = 2.39-28.14) in the participants who were NC at the baseline, and 8.41 (95% CI = 4.05-17.50) in those who were MCI at the baseline. The HR for 2-year mortality of the incident dementia cases was reduced to about 5.53 (95% CI 3.06-9.99) when we employed the non-demented people, i.e., people with stable NC, prevalent or incident MCI, as the reference group. Compared to the participants with stable NC, the participants with incident MCI and stable MCI showed about a 2-fold increased risk of 2-year mortality. However, the mortality of the participants with reverted MCI was comparable to that of the participants with stable NC (Table 3).

**Table 3:** The risk of mortality according to the diagnosis at baseline and 2-year follow-up assessment.

Diagnosis	No. of participants	Deaths		
		Person-years	No.	HR (95% CI)*
NC at both baseline and follow-up	3079	5406.6	28	1.00
Prevalent MCI†	744	1285.0	23	1.94 (1.06 - 3.52)
Incident MCI‡	502	848.5	17	2.22 (1.14 - 4.30)
Prevalent dementia§	150	251.8	13	2.82 (1.28 - 6.22)
Incident dementia§§	95	158.5	15	8.37 (4.23 - 16.54)
Not demented at both baseline and follow-up	4849	8494.1	79	1.00
Prevalent dementia§	150	251.8	13	1.82 (0.90 - 3.69)
Incident dementia§§	95	158.5	15	5.53 (3.06 - 9.98)

HR = hazard ratio; CI = confidence interval; NC = normal cognition; MCI = mild cognitive impairment

* Adjusted by age, educational level, sex, alcohol consumption, smoking status, Cumulative Illness Rating Scale score, Modified Hachinski Ischemic Score, and the presence of a depressive disorder

† MCI in both the baseline and follow-up assessments

‡ NC in the baseline assessment and converted to MCI in the follow-up assessment

§ Dementia in both the baseline and follow-up assessment

§§ Not demented in the baseline assessment but was converted to dementia in the follow-up assessment

Compared to the participants with stable NC, those with incident dementia showed the least favorable rates of survival (log-rank test = 161.326, p < 0.001) followed by those with prevalent dementia (log-rank test = 71.982, p < 0.001). The participants with prevalent and incident MCI showed less favorable rates of survival than those with stable NC or reverted MCI (log-rank test = 22.318 and 22.207, p < 0.001), but more favorable rates of survival than those with incident dementia (log-rank test = 23.593 and 31.515, p < 0.001) or prevalent dementia (log-rank test = 7.174 and 10.200, p = 0.007 and 0.001) (Fig. 3).

## DISCUSSION

This study showed that the ROM in the elderly with newly incident dementia was higher than the ROM in those with previously diagnosed dementia, and incident dementia increased the ROM of elderly people more than previously reported. To our knowledge, no study has directly compared the ROM of dementia between the prevalent and incident cases in a population. As summarized in Table 4, the HRs for mortality reported in incident dementia cases ranged from 1.7 to 3.1 [13-18], which were rather at the lower end of the scale when compared to the HRs reported in studies which enrolled prevalent cases (2.2-6.3) [2-12] in previous studies. Guhne et al.[16] assumed that the studies using prevalent cases for estimating mortality were biased in so far as they included more severe cases. However, in this study, the 2-year HR for mortality in the incident dementia cases was about 3 times higher than that in prevalent dementia cases, despite the fact that the severity of dementia, in the incident cases, was milder than that in the prevalent cases.

These results directly showed that the estimation of the ROM in dementia may be subject to length bias. Length bias can lead to an underestimation of the deleterious effects of diseases because of the failure to include persons with rapidly progressive diseases, who died before they could be included in the study [26, 27]. In a population-based cohort study in Netherlands, 8-13% of the dementia patients died within 2 years after diagnosis and their mean survival was 12 months [41]. In the dementia cases from the Canadian Study of Health and Aging, the estimated median survival was markedly reduced, from 6.6 years to 3.3 years, when the length bias was adjusted [27]. The studies using incident cases may also have been subject to length bias. Some participants with rapidly progressive diseases may progress to dementia during the follow-up period and die before follow-up assessment. The population-attributable risks (PARs) of AD, estimated using incident cases in 3 previously conducted studies, were different according to the intervals of the follow-up assessments. The PARs of AD were estimated to be 37% in a study which conducted annual follow-ups [14], while the value was 18.3% in a study with biannual follow-ups and 15% in a study with triannual follow-ups [15, 17].

**Table 4:** Studies investigating the risk of mortality of dementia or Alzheimer’s disease.

Study (year of publication)	Number	Age (years)	Sex (F, %)	Diagnosis	Reference group	HR of dementia (or AD)	Duration of follow-up	Follow-up assessment	Interval of follow-up assessment (years)
Aguero-Torres et al. (1998)	989	77+	77	Incident dementia	Non-demented	2.0 (1.5-2.7)	5.0	Yes	3.4
Aevarsson et al. (1998)	494	95+	71	Prevalent dementia, AD	Non-demented	2.6 (male) 2.9 (female)	7.0	No	
Baldereschi et al. (1999)	5632	65+	49	Prevalent dementia	Non-demented	3.56 (2.52-5.04)	2.3	No	
Helmer et al. (2001)	3777	65+		Incident dementia	Non-demented	1.80 (1.46-2.21)	8.0	Yes	2.2
Noale et al. (2003)	5632	65+	49	Prevalent dementia	Non-demented	3.72 (3.01-4.60)	4.0	No	
Tschanz et al. (2004)	4683	65+	57	Prevalent dementia	Non-demented	2.99 (2.53-3.53)	5.0	No	
Fitzpatrick et al. (2005)	3602	65+	59	Incident dementia, AD	Non-demented	Dementia: 2.8 (2.3-3.4) AD: 2.1 (1.6-2.7)	6.5	Yes	1.0
Nitrini et al. (2005)	1956	65+		Prevalent dementia	Non-demented	3.92 (2.80-5.48)	4.0	No	
Ganguli et al. (2005)	1681	65+	58	Prevalent AD + Incident AD	Non-demented	1.7 (1.4-2.0)	10.3	Yes	2.0
Guhne et al. (2006)	1124	75+	75	Incident dementia	Non-demented	2.42 (1.62-3.63)	4.5	Yes	1.6
Scarmeas et al. (2007)	338	65+	78	Prevalent AD	Non-demented	2.38 (1.86-3.04)	4.4	No	
Llinàs-Regla et al. (2008)	1153	70+	57	Prevalent dementia	Non-demented	2.3 (1.7-3.2)	4.3	No	
Wilson et al. (2009)	1715	65+	62	Prevalent AD	Normal cognition	2.84 (2.29-3.52)	4.7	No	
Villarejo et al. (2011)	5262	65+	58	Prevalent dementia	Non-demented	3.16 (2.74-3.65)	13	No	
Wu et al. (2011)	2788			Prevalent dementia	Non-demented	2.18 (1.75-2.71)	7.3	No	
James et al. (2014)	2566	65+	72	Incident AD	Non-demented	3.13 (2.74-3.58)	8.0	Yes	1.0
Chen et al. (2014)	2978	60+		Prevalent dementia	Non-demented	2.69 (2.11-3.42)	5.0	No	
Park et al. (2014)	1035	65+	58	Prevalent dementia	Normal cognition	3.20 (2.30-4.44)	8.0	No	
Paddick et al. (2015)	1198	70+	71	Prevalent dementia	Normal cognition	6.33 (3.19-12.58)	4.0	No	

HR = hazard ratio; AD = Alzheimer’s disease; F= female

In the present study, the 2-year HR of the mortality of the incident dementia cases was 8.37, which was much higher than those estimated in previously conducted studies [13-18]. Since the 2-year HR of mortality of the prevalent dementia cases, estimated in the present study, was comparable to those estimated in other studies [2-12], the high estimated HR of the incident dementia cases could be attributed to the following reasons, rather than the differences in the study samples, between the present and previous studies. First, 6 previous studies on the ROM of incident dementia cases employed non-demented individuals as their reference groups, instead of individuals with NC. The enrollment of individuals with MCI, who were potentially included in the non-demented control groups, might have reduced the estimated HR of mortality in those studies [13-17]. In the present study, the HR of mortality of the incident dementia cases reduced from 8.37 to 5.53 when both NC and MCI cases were included in the reference group. Second, the duration of the follow-ups in the previous studies was longer than that in the current study. Of the 6 studies which enrolled incident dementia cases, the average follow-up duration was 4-8 years in 3 studies [16-18] and over 8 years in the rest [13-15]. As the duration of the follow-up increases, the proportion of dementia cases with longer survival times after dementia onset, may increase; thus, the estimated ROM may decrease.

This study did not directly investigate why newly incident dementia had high mortality. However, the participants with incident dementia had higher CIRS scores at baseline than those with prevalent dementia. In future studies, it should be investigated whether comorbid medical illnesses that may increase mortality may accelerate the progression of dementing illness from preclinical stage to clinical stage, or the speed of cognitive decline may result in the different ROM between prevalent and incident dementia. Rate of cognitive decline was strongly associated with the ROM in AD patients [42]. Because the participants who developed dementia within 2 years were classified as incident dementia in the present study, they might have faster cognitive decline than those with prevalent dementia. There are risk factors that increased both dementia and mortality risks. Smoking increased risk of incident AD and vascular dementia [43], and it also elevated ROM from all cause, cardiovascular disease and cancer [44]. Medical illnesses such as cardiovascular disease and diabetes increased the risk of conversion from MCI to dementia [45] as well as the ROM [46]. Depression is closely related with the development of dementia [47], and high levels of depressive symptoms were risk factor for mortality in the elderly [48]. However, these factors were adjusted in our Cox proportional hazard model. Physicians may need to inform the caregivers or family members of the people with newly incident dementia that the patients may not be able to manage pre-existing chronic disease as properly as before.

This study showed that the ROM of MCI was not susceptible to length bias; the HR of mortality of both incident and prevalent MCI cases was about 2. Only 1 other study reported the ROM in both incident and prevalent MCI cases. In that study, the HR of mortality was also about 2, in both prevalent and incident MCI cases [22].

This study found that newly incident dementia patients were high risk group for mortality, and their ROM was higher than those with dementia for a certain period of time.

This study has several strengths worth mentioning. This study was conducted on a large, randomly sampled, nationwide elderly population, and mortality was identified by the National Mortality Database, which provides reliable information on mortality and encompasses 100% of the Korean population. Therefore, the results of this study could be generalizable to the Korean elderly population. In addition, all the participants were evaluated by geropsychiatrists, who were experts in dementia research, using structured and standardized clinical and neuropsychological evaluations. Potential confounding factors were rigorously adjusted in the Cox proportional hazard models. However, there are limitations in this study. First, some information such as chronic illness was obtained by self-report of participant and/or informants. Therefore, this information might not be accurate, and some participants might not recognize their disease. Second, although we adjusted potential confounding factors in analysis, there might be other confounding factors that affect the association between dementia and mortality. Third, we did not adjust for the influence of apolipoprotein E genotype because this was assessed in only some of the participants. Fourth, this study did not compare the causes of death between the diagnostic groups. Fifth, the total duration of follow-up was too short to estimate the disease-specific median survivals. The median survival time after dementia onset ranged from 3.1 to 5.9 years, in other studies [13-17].

The ROM due to dementia may be much higher than previously expected. Considering that dementia is often omitted as an underlying cause of death, on death certificates [14], the number of deaths attributable to dementia may be much higher than that reported on death certificates. In clinical settings, the ROM warrants the attention of physicians, particularly in recently incident dementia cases.
